# A Systematic Review of Tissue Engineering Scaffold in Tendon Bone Healing *in vivo*

**DOI:** 10.3389/fbioe.2021.621483

**Published:** 2021-03-15

**Authors:** Zimu Mao, Baoshi Fan, Xinjie Wang, Ximeng Huang, Jian Guan, Zewen Sun, Bingbing Xu, Meng Yang, Zeyi Chen, Dong Jiang, Jiakuo Yu

**Affiliations:** ^1^Sports Medicine Department, Beijing Key Laboratory of Sports Injuries, Peking University Third Hospital, Beijing, China; ^2^Institute of Sports Medicine of Peking University, Beijing, China; ^3^School of Clinical Medicine, Weifang Medical University, Weifang, China; ^4^Qingdao University, Qingdao, China; ^5^Department of Sports Medicine, The Affiliated Hospital of Qingdao University, Qingdao, China

**Keywords:** bone tissue engineering, scaffold, biomaterials, tendon bone healing, review

## Abstract

**Background:** Tendon-bone healing is an important factor in determining the success of ligament reconstruction. With the development of biomaterials science, the tissue engineering scaffold plays an extremely important role in tendon-bone healing and bone tissue engineering.

**Materials and Methods:** Electronic databases (PubMed, Embase, and the Web of Science) were systematically searched for relevant and qualitative studies published from 1 January 1990 to 31 December 2019. Only original articles that met eligibility criteria and evaluated the use of issue engineering scaffold especially biomaterials in tendon bone healing *in vivo* were selected for analysis.

**Results:** The search strategy identified 506 articles, and 27 studies were included for full review including two human trials and 25 animal studies. Fifteen studies only used biomaterials like PLGA, collage, PCL, PLA, and PET as scaffolds to repair the tendon-bone defect, on this basis, the rest of the 11 studies using biological interventions like cells or cell factors to enhance the healing. The adverse events hardly ever occurred, and the tendon bone healing with tissue engineering scaffold was effective and superior, which could be enhanced by biological interventions.

**Conclusion:** Although a number of tissue engineering scaffolds have been developed and applied in tendon bone healing, the researches are mainly focused on animal models which are with limitations in clinical application. Since the efficacy and safety of tissue engineering scaffold has been proved, and can be enhanced by biological interventions, substantial clinical trials remain to be done, continued progress in overcoming current tissue engineering challenges should allow for successful clinical practice.

## Impact Statement

The poor healing of tendon and bone is an important problem that puzzles the postoperative recovery of ligament reconstruction. Usually, after arthroscopic surgery, there is a gap between the graft and bone healing in the bone tunnel. Therefore, researchers hope to obtain better healing effect through other ways such as tissue engineering, so as to improve the function recovery of ligament and joint postoperatively. In this paper, a systematic summary of the *in vivo* research was carried out, hoping to summarize the research achievements and find new inspiration and breakthrough to inspire future research.

## Introduction

Tendon defect is one of the common clinical diseases (Hayashida et al., 2018; Kunze et al., 2019). After the tendon injury, it often leads to limb dysfunction, severe cases, or even disability without repairation in time (Ando et al., 2019). Tendon-bone healing is an important factor in determining the success of ligament reconstruction (Li et al., 2019; Huang et al., 2020). Whether it is knee cruciate ligament, lateral collateral ligament, posterolateral complex reconstruction, shoulder joint sleeve repair, or ankle ligament reconstruction, the degree of tendon-bone healing directly affects the postoperative rehabilitation process and surgical effect (Zhao et al., 2019). Studies have shown that in addition to surgical errors, failure to achieve strong tendon-bone healing is the main reason for failure of ligament reconstruction surgery (Han L. et al., 2019; Zhang et al., 2020). Tendon injuries can be divided into two categories: non-defective injuries and defective injuries (Van Der Made et al., 2019; Wisbech Vange et al., 2020). For the tendons with defects and injuries, several treatment methods are available, such as autogenous tendon transplantation, allogeneic tendon transplantation, heterogeneous tendon transplantation, and artificial tendon replacement (Periasamy et al., 2019). In the former, due to lack of donor tendon or immune rejection, the tendon transplantation is restricted. With the development of cell culture technology and transplantation technology and the development of biomaterials science, a new ideal tendon replacement-tissue engineered artificial tendon with synthetic materials, will eventually solve the problem of repairing defective tendons (Khoo and Nikkhah, 2019).

Tissue engineering scaffold mainly serves the following aspects: (1) As a framework connecting cells and tissues, it can be used to guide tissues to grow into a specific shape (Lewandowska-Lancucka et al., 2015). (2) As a carrier of signaling molecules, it is transported to the defect site, and as a slow-release body, the osteoinductive factor slowly acts. (3) As a place for bone tissue to reproduce, differentiate and metabolize, transport nutrients for cell growth, and eliminate waste. (4) The specific sites on the surface of the scaffold react specifically with the cells and play the role of “identification” and selective adhesion to different types of cells. It can be seen that the scaffold plays an extremely important role in bone tissue engineering, which not only plays a physical role in connecting and supporting cells and tissues but also regulates various functional activities of cells. Tendon/bone tissue engineering has high requirements for scaffold materials, and an ideal *in vivo* graft should meet the following points. For example, the scaffold materials must be non-toxic and have good biocompatibility (Karel et al., 2018). The material must also be biodegradable and can be gradually degraded and metabolized in the body as the cells proliferate, and then be absorbed (Hejbol et al., 2017). Besides, the degradation products of the materials must be non-toxic, with good biocompatibility, and will not adversely affect tissues and organisms. In addition, the material must have good processing properties and can be processed into the required shape and structure like open-pore structure and proper pore size. The scaffold must have good cell affinity, suitability for cell adhesion, proliferation, and secretion of the matrix, as well as certain mechanical properties, including strength and flexibility. Under conventional sterilization conditions, the scaffold must be able to withstand sterilization without physical, chemical, and biological changes. Moreover, the scaffold should not only maintain its shape during the cell culture operation but also be able to withstand the surgical operations implanted in the body, to ensure that it will not break during the operation, can fit with the body, and will not cause mechanical damage to the body tissue.

In recent years, studies on improving tendon-bone healing have mainly focused on promoting ligament-bone integration at tendon sites (Xu et al., 2019). This includes the use of periosteum, biogels, scaffolds, growth factors, stem cells, or other reconstruction materials that promote bone growth or ligament attachment points (Uz et al., 2019; Huang et al., 2020; Rodriguez-Vazquez and Ramos-Zuniga, 2020; Sadeghinia et al., 2020). However, reviews describing the application of tissue engineering scaffold, especially biomaterials, in tendon-bone healing *in vivo* are lacking. The purpose of this systematic review was to (1) critique the evidence *in vivo* regarding the use of tissue engineering scaffold to achieve tendon-bone healing; (2) provide a descriptive summary of the current evidence for tissue engineering scaffold use in tendon-bone healing especially biomaterials, as this is the first systematic review on the topic; and (3) highlight areas of future research to facilitate clinical application.

## Methods

### Eligibility Criteria

This systematic review was conducted according to the Preferred Reporting Items for Systematic Reviews and Meta-Analyses (PRISMA) guidelines (Maher et al., 2003). This review included original peer-reviewed studies based on the following inclusion criteria: (1) publication in an English-language journal; (2) an *in vivo* animal study or clinical study that mainly evaluated the use of issue engineering scaffold in tendon bone healing; and (3) scaffold which was manufactured using biomaterials. Studies reporting *in vitro* work without *in vivo* analysis were excluded. Only original peer-reviewed articles were included, so letters, editorials, review articles, conference, patents, and meeting abstracts and studies not involving tissue engineering scaffold were excluded.

### Literature Search Strategy

Literature searches were conducted in three electronic databases (PubMed, EMBASE, and Web of Science) from 1 January 1990 to 31 December 2019. The following search terms were used for the literature search: “(tissue engineering OR tissue engineering scaffold OR scaffold for tissue engineering OR scaffold) AND (tendon-bone healing OR tendon-bone OR healing of bone-tendon) AND (*vivo* OR human OR patient OR animal OR mouse OR rat OR rabbit OR dog OR sheep OR pig OR horse OR ovine).” Because the scope of this review was large in terms of outcome measures, a systematic review, not a meta-analysis, was performed.

### Study Selection

The articles were initially screened to assess suitability for inclusion according to the criteria with their title and abstract, and then the full text of each article was downloaded to define the relevance of the work for investigators. During selection, the following information was extracted: details of the animal model or clinical information, the groups investigated, the types of scaffold, the methods of evaluation, and the main findings. The selected articles in the study were reviewed, evaluated, and discussed by the authors. A senior investigator would make the final decision if the reviewers were not able to reach a consensus agreement on the inclusion of any articles.

## Results

### Search Results

**Figure 1:**
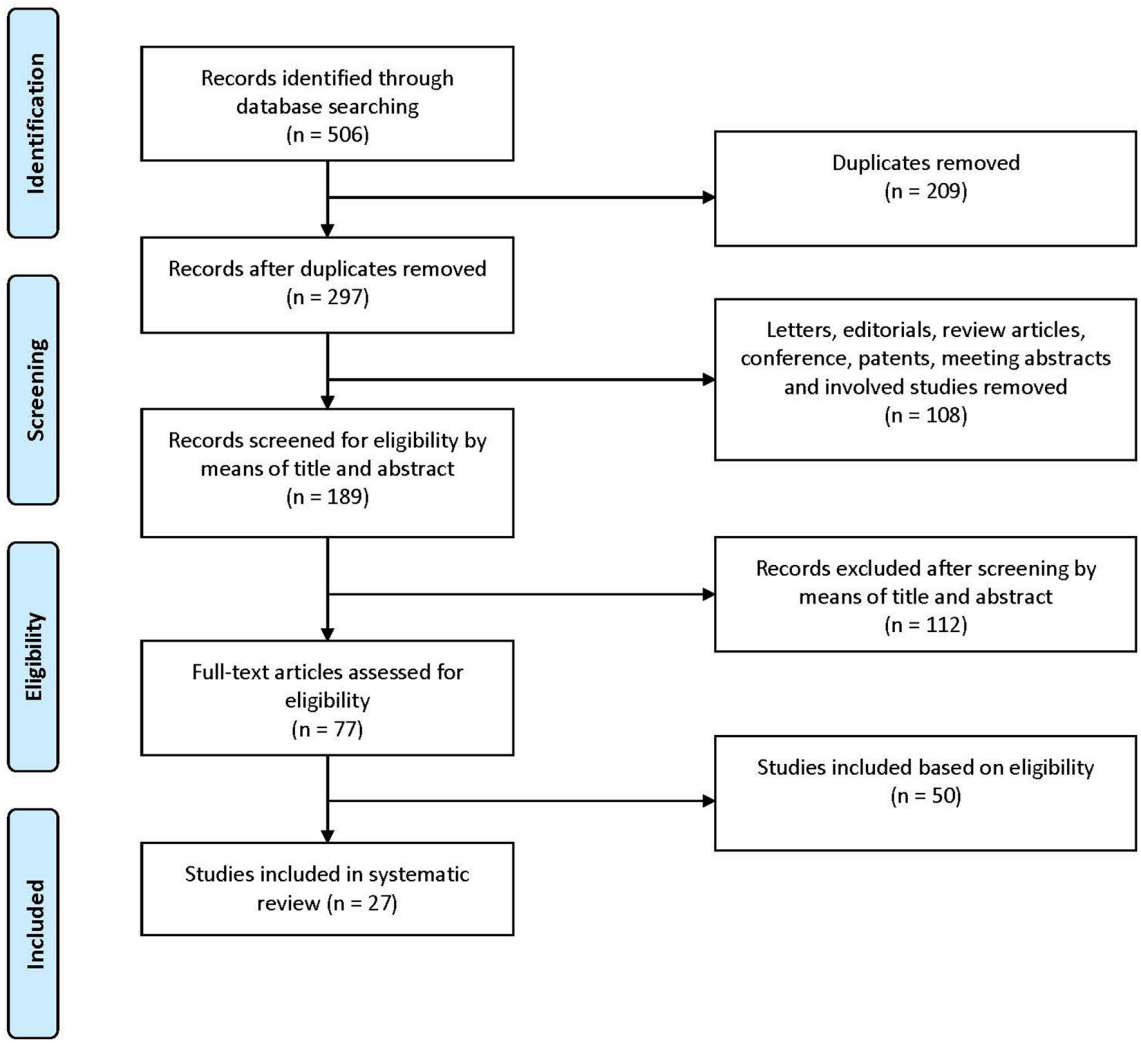
A flowchart showing the selection of studies for inclusion in the systematic review.

Figure 1 represents the process for evaluating studies for inclusion in the systematic review. A total of 506 articles were identified through our search. After removal of duplicates, as well as letters, editorials, review articles, conference, patents, meeting abstracts, and involved studies, 189 articles were screened for eligibility by means of title and abstract. Following the exclusion of 112 articles, 77 full-text articles were assessed for eligibility. Finally, 27 studies were identified and included in our analysis, which were consisted of two human trials and 25 animal studies. Furthermore, the 27 studies using this retrieval strategy were analyzed.

### Data Extraction

Information of data extraction of the 27 studies is available in Table 1, covering details of the animal model or clinical information, the groups investigated, and the types of scaffold. Of the 27 included *in vivo* studies, two were human trials (Petruskevicius et al., 2002; Mochizuki and Ochi, 2015) and 25 were animal studies. More than half of the animal studies (*n* = 14) utilized a rabbit model (Zhang et al., 2011; Li et al., 2012, 2017; Jiang et al., 2014; Han et al., 2015; Chou et al., 2016; Chung et al., 2017; Lee et al., 2017; Cai et al., 2018; Hu et al., 2018; Chen P. et al., 2019; Han F. et al., 2019; Learn et al., 2019), eight studies used rats (Loeffler et al., 2013; Zhao et al., 2014, 2015a,b; Kovacevic et al., 2015; Zhang et al., 2018; Zhu M. et al., 2019; Zhu Q. et al., 2019), and three studies used pigs (Fleming et al., 2009; Vavken et al., 2012; Li et al., 2014). The involved patients and animals were mature, and no minor or immature animal model had been investigated in previous studies so far as searched. Among the two human trials, only one had tibia defect, which was repaired with Osteoset (Petruskevicius et al., 2002), the other had not point out the defective part (Mochizuki and Ochi, 2015). In animal models, the defect occurred in supraspinatus tendon, infraspinatus tendon, anterior cruciate ligament, long digital extensor tendon, achilles tendon, and proximal tibial metaphysis. Moreover, there were 15 studies that only used synthetic scaffolds like poly lactide-co-glycolide (PLGA), collage, polycaprolactone (PCL), polylactic acid (PLA), and polyethylene terephthalate (PET) to repair the defect of tendon-bone interface, and the rest of the 12 studies used biological interventions, which combined cells or cell factors to enhance the tendon bone healing, such as bone morphogenetic protein (BMP), stromal cell-derived factor (SDF), ligament-derived stem/progenitor cells (LSPCs), kartogenin (KGN), platelet-derived growth factor (PDGF), mesenchymal stem cell (MSC), platelet, etc.

**Table 1:** The information of data extraction of the included studies.

	**References**	**Species**	**Age of animals orpatients**	**Weight of animals or patients**	**Type of scafford**	**Biological intervention**	**Group**	**Defect parts**
Human studies	Mochizuki and Ochi, 2015	Human	65.5 years (range: 57–77 years) old	N/A	PGA sheet	None	PGA group (*n* = 30); PG group (*n* = 32);	N/A
	Petruskevicius et al., 2002	Human	19–44 years	N/A	Osteoset	None	Osteoset group (*n* = 10); Control group (*n* = 10)	Tibia
Animal studies	Chen C. et al., 2019	Rabbit	Adult	2.00–2.20 kg	PLGA fibrous scaffold	BMP-12	Control group (*n* = 8); Lenti-BMP (bone morphogenetic proteins)-12 group (*n* = 8)	Supraspinatus tendon
	Learn et al., 2019	New Zealand rabbit	8–13 months old	3–5 kg	Electrochemically aligned collagen (ELAC)	MSC	Direct repair (*n* = 5); Scaffold-alone (*n* = 5); Cell-seeded scaffold repair (*n* = 5)	Infraspinatus tendon in the right shoulder
	Zhu M. et al., 2019	Sprague-Dawley rat	≥12 weeks old	≥350 g	Synthetic collagen	None	Sham surgery group (*n* = 6); Unaugmented control group (*n* = 20); Intervention group (*n* = 20)	Supraspinatus tendon
	Han F. et al., 2019	New Zealand rabbit	12 weeks old	2.4 ± 0.3 kg	PCL scaffold	BMP-2; SDF-1α	PCL group (*n* = 6); B@P group (PCL scaffold loaded with BMP-2) (*n* = 6); S + B@P (PCL scaffold loaded with BMP-2 and SDF-1α) (*n* = 6)	Anterior cruciate ligament of the right knee joint
	Zhu Q. et al., 2019	Sprague-Dawley rat	2 months old	400–500 g	PCL fibrous membranes with aligned nanofibers	KGN	Control group (n = 45); PCL group (n = 45); KGN-PCL group (PCL scaffold loaded with KGN)(n = 45)	Supraspinatus tendon (superior of the scapula)
	Zhang et al., 2018	Mature female Sprague-Dawley rats	N/A	200–220 g	PLLA aligned fiber (A-TSA) scaffold	Trichostatin A	A-TSA group (*n* = 7) A groups (*n* = 7) R-TSA group (*n* = 7) R group (*n* = 7)	Achilles tendon
	Cai et al., 2018	New Zealand rabbit	6–8 months old	2.8–3.2 kg	SF/P(LLA-CL) nanofibrous scaffolds using silk fibroin (SF)-blended poly(L-lactic acid-co-e-caprolactone) (PLLA-CL)	None	Random scaffold (RS) (*n* = 30); Dual-layer aligned-random nanofibrous scaffolds(ARS) (*n* = 30); Control groups (*n* = 30)	Achilles tendon of one hindlimb
	Hu et al., 2018	New Zealand rabbit	N/A	2.5–3.0 kg	Collage-silk; SDF-1-releasing collagen-silk (CSF) scaffold	SDF-1; LSPCs	CS (collage-silk) (*n* = 10); CSL (collage-silk with LSPCs injection) (*n* = 15); CSF (collage-silk-SDF-1) (*n* = 10); CSFL (collage-silk-SDF-1 with LSPCs injection) (*n* = 15);	Anterior cruciate ligament of the right knee joint
	Li et al., 2017	New Zealand rabbit	6–8 months old	2.8–3.5 kg	Simplex fibrous membrane of PLLA; Dual-layer organic/inorganic flexible bipolar fibrous membrane (nHA-PLLA)	None	Control group (*n* = 48); Simplex fibrous membrane of PLLA (SFM) group (*n* = 48); dual-layer organic/inorganic flexible bipolar fibrous membrane (BFM) group (*n* = 48)	Supraspinatus tendon
	Chung et al., 2017	New Zealand rabbit	6–7 months old	3.2–3.5 kg	A biodegradable and synthetic tri-component graft consisting of porous poly(1,8-octanediol-co-citric acid)–hydroxyapatite nanocomposites (POC–HA) and poly(l-lactide) (PLL) braids	None	Reconstructed animals (*n* = 3); Non-operated control animals (*n* = 3)	Anterior cruciate ligament of the right knee joint
	Lee et al., 2017	New Zealand rabbit	Adult	3.0–3.5 kg	Recombinant human rhBMP-2-containing collagen gel	rhBMP-2	Saline only (control) (*n* = 12); Collagen gel without rhBMP-2 (*n* = 12); rhBMP-2-conjugated collagen gel (*n* = 12)	Long digital extensor tendon
	Chou et al., 2016	New Zealand rabbit	Adult	3.3 ± 0.7 kg	PLA; PLGA/collagen nanofibrous membrane	None	Control (*n* = 24); PLGA/collagen nanofibrous membrane (*n* = 24)	Long digital extensor tendon
	Han et al., 2015	New Zealand rabbits	12 weeks old	2.4 ± 0.3 kg	A biomimetic nanofiber membrane of polycaprolactone/ nanohydroxyapatite/collagen (PCL/nHAp/Col)	None	PCL/nHAp/Col group (*n* = 6); PCL group (*n* = 6)	Anterior cruciate ligament of the right knee joint
	Bi et al., 2015	New Zealand rabbit	13 weeks old	2.5–3.0 kg	Silk-collagen scaffold	None	Scaffold group (*n* = 20); Autograft group (*n* = 20)	Anterior cruciate ligament of the right knee joint
	Kovacevic et al., 2015	Sprague-Dawley rat	N/A	N/A	Collagen scaffold	PDGF	Control (*n* = 19); Scaffold only (*n* = 19); Three PDGF doses (30 μg/mol/L, 100 μg/mol/L, 300 μg/mol/L) on the collagen scaffold (*n* = 19)	Supraspinatus tendon
	Zhao et al., 2015a	Sprague-Dawley rat	N/A	350–400 g	PCL scaffold; Scaffold composed of microfibers of PCL and nanofibers of CS	None	Transosseous repair (*n* = 48); PCL scaffolds (*n* = 48) PCL-CS scaffolds (*n* = 48)	Left supraspinatus tendon
	Zhao et al., 2015b	Sprague-Dawley rat	N/A	350–401 g	PLLA fibrous membranes; Gelatin-PLLA	None	Transosseous repair (*n* = 48); PLLA membranes (*n* = 48); Gelatin-PLLA membranes (*n* = 48)	Left supraspinatus tendon
	Li et al., 2014	Pig	3 months old	47 and 52 kg	Silk–TCP–PEEK scaffold of silk, TCP, and PEEK	None	N/A	Anterior cruciate ligament of the knee joint
	Jiang et al., 2014	New Zealand rabbit	Adult	2.8 ± 0.5 kg	PET	None	PET group (*n* = 24); PET + SF group (SF-coating group) (*n* = 24); PET + SF + HAP group (combined HAP- and SF-coating group) (*n* = 24)	Proximal tibial metaphysis
	Zhao et al., 2014	Sprague-Dawley rat	N/A	350–400 g	PLGA membranes	bFGF	Transosseous repair (*n* = 48); PLGA membranes (*n* = 48); bFGF–PLGA membranes (*n* = 48)	Left supraspinatus tendon
	Loeffler et al., 2013	Lewis rat	N/A	13.8 weeks ± 2/7	Sponge	Cultured cells from the tendon-to-bone interface	Group I (control without surgery); Group II (surgical defect in the rotator cuff only); Group III (surgical defect with suture repair only); Group IV (surgical defect and repair with sponge only); Group V (surgical defect and repair with sponge loaded with cells)	Supraspinatus tendon
	Vavken et al., 2012	Yorkshire pig	11.8 ± 0.4 weeks old	30 ± 1.1 kg	Collagen Scaffold	Platelet	ACL reconstruction (*n* = 8); Enhanced ACL repair (*n* = 8); ACL transection (*n* = 8);	Anterior cruciate ligament of the knee joint
	Li et al., 2012	New Zealand rabbit	Adult	N/A	PET artificial ligament	None	Control group; LBL group	Anterior cruciate ligament of the knee joint
	Zhang et al., 2011	New Zealand rabbit	22 weeks old	2.7 ± 0.2 kg	Fibrin glue-BMP	BMP	Control group (*n* = 17); Fibrin glue-BMP group (*n* = 17); RBX (recombined bone xenograft) group (*n* = 17)	Long digital extensor tendon
	Fleming et al., 2009	Yorkshire pig	N/A	30 kg	Collagen-platelet composite (CPC)	None	Control group (*n* = 7); Experimental Group (*n* = 7)	Anterior cruciate ligament of the knee joint

*PGA, polyglycolic acid; BMP, bone morphogenetic protein; PCL, polycaprolactone; SDF, stromal cell-derived factor; PDGF, platelet-derived growth factor; PLLA, poly l-lactide; TCP, tricalcium phosphate; ELAC, electrochemically aligned collagen; PLA, polylactic acid; SF, silk fibroin; PLGA, poly lactide-co-glycolide; MSC, mesenchymal stem cell; KGN, kartogenin; LSPCs, ligament-derived stem/progenitor cells; CS, chitosan; PET, polyethylene terephthalate; PEEK, polyether ether ketone; ACL, anterior cruciate ligament; HAp/HA, hydroxyapatite*.

### Safety and Efficacy Evaluation

The adverse events, histological and biomechanical results, and data from other tests were assessed to determine the scaffold safety and efficacy (Table 2). As for the adverse events, no complications were encountered; all the participants and animals recovered well, and macroscopic inspection of the collected specimens did not reveal any gross infectious or inflammatory changes in most of the included studies *in vivo*. Only one rabbit with self-inflicted wound developed dehiscence and heterotopic of bone formation in the study of Learn et al. (2019), and one animal with minor damage upon tricalcium phosphate (TCP) insertion was found in the study of Li et al. (2014). As for the histological results, it was demonstrated that the tendon-bone integration at the interface using synthetic scaffolds was better than transosseous or direct repair and could be enhanced by biological interventions. The healing was generally characterized by collagen organization improvement, glycosaminoglycan deposition, new bone formation (mineralization), fibroblast-like cells, chondrocyte-like cells, and immature neo-enthesis structure increasement. As for biomechanical tests, the results of ultimate force to failure, stiffness, and strength were extensively investigated and compared, which suggested that the tendon-bone healing with scaffolds produced superior biomechanical outcomes to transosseous or direct repair, especially combined with biological interventions. In addition, other tests were performed to evaluate the efficacy of tissue engineering scaffold in tendon bone healing, such as micro-CT, JOA score, and mRNA level, whose results were consistent with the histological results.

**Table 2:** The safety and efficacy of tissue engineering scaffold in tendon bone healing *in vivo*.

	**References**	**Group**	**Follow-up**	**Adverse events**	**Histological data**	**Biomechanical data**	**Data from other tests**
**Human studies**	Mochizuki and Ochi, 2015	PGA group (*n* = 30); PG group (*n* = 32);	1, 3, 6, 12 months	None	N/A	N/A	The mean JOA scores of PGA group and PG group improved and the high-intensity rate was significantly lower for the PGA group by MRI
	Petruskevicius et al., 2002	Osteoset group (*n* = 10); Control group (*n* = 10)	6 weeks, 3, 6 months	None	N/A	N/A	The same amount of bone in the defect was found in the Osteoset and control groups, but the bone volume increased in the control group, and the Osteoset pellets were almost resorbed after 6 weeks
**Animal studies**	Chen C. et al., 2019	Control group (*n* = 8); Lenti-BMP (bone morphogenetic proteins)-12 group (*n* = 8)	4, 8, 12 weeks	N/A	Application of BMP-12 overexpressing BM-MSCs-loaded PLGA scaffolds increased the amount of fibrocartilage formation and improve the collagen fiber organization at the interface between the tendon and bone	The ultimate force to failure in the Ad-BMP-12 group was significantly higher than that in control group	The Ad-BMP-12 group had significantly higher modified tendon maturing scores than the control group at 4, 8, and 12 weeks post-surgery
	Learn et al., 2019	Direct repair (*n* = 5); Scaffold-alone (*n* = 5); Cell-seeded scaffold repair (*n* = 5)	3 months	One rabbit with self-inflicted wound dehiscence and heterotopic bone formation	Robust collagen deposition around ELAC fibers and increased cellularity within the continuum of woven scaffolds as compared to native tendon	The maximum load-bearing capacity was comparable between all groups, while MSC-seeded scaffold repairs exhibited increased stiffness relative to non-seeded scaffold repairs	Immunohistochemical staining revealed presence of collagens I and III in all groups, but procollagen I and the tendon-specific marker tenomodulin were only observed in seeded and non-seeded ELAC scaffold repairs
	Zhu M. et al., 2019	Sham surgery group (*n* = 6); Unaugmented control group (*n* = 20); Intervention group (*n* = 20)	6, 12 weeks	None	Improved collagen fiber density and orientation scores in the tendon and improved enthesis with early formation of a fibrocartilage transition zone were observed in the scaffold group	No significant difference was detected in Biomechanical analysis	N/A
	Han F. et al., 2019	PCL group (*n* = 6); B@P group (PCL scaffold loaded with BMP-2) (*n* = 6); S + B@P (PCL scaffold loaded with BMP-2 and SDF-1α) (*n* = 6)	4, 8 weeks	N/A	Thin fibrous scar tissue in the tendon-bone interface and a large amount of new bone inside the autograft were formed in the S + B@P group. In the B@P group, the fibrous scar tissue was thin, and there was less new bone formation at the interface. In the PCL group, there was still more fibrous scar tissue at the interface, no significant mineralization was observed, and many membranes remained.	The S + B@P group exhibited superior mechanical properties compared to those the B@P and PCL groups, and had a higher failure force and stiffness	Immunohistochemical staining revealed that the secretion of OCN and OPN in the bone tunnel always remained at a high level in the S + B@P group
	Zhu Q. et al., 2019	Control group (*n* = 45); PCL group (*n* = 45); KGN-PCL group (PCL scaffold loaded with KGN) (*n* = 45)	2, 4, 8 weeks	N/A	KGN-PCL membranes promoted fibrocartilage formation and collagen organization	The ultimate load to failure in the PCL-KGN group was highest among the three groups, and that in PCL group was higher than that in the control group	N/A
	Zhang et al., 2018	A-TSA group (*n* = 7) A groups (*n* = 7) R-TSA group (*n* = 7) R group (*n* = 7)	2, 4 weeks	N/A	The formation of regenerated tendon with the typical structure of tendon at the repaired site in the A-TSA and A treated groups. R-TSA treated group had a much better histological structure compared with R treated group.	The A-TSA group had better biomechanical properties than other three groups	The mRNA expression of Mkx was upregulated ~2.3-fold in A-TSA treated group. The average diameter of collagen fibrils in the A-TSA group (50.48 ± 10 nm) was larger than other groups.
	Cai et al., 2018	Random scaffold (RS) (*n* = 30); Dual-layer aligned-random nanofibrous scaffolds(ARS) (*n* = 30); Control groups (*n* = 30)	6, 12 weeks	N/A	The ARS significantly increased the area of metachromasia, decreased the interface width, and improved collagen maturation and organization at the tendon–bone interface compared with the RS and control groups	The ARS groups had a better ultimate load-to-failure and stiffness than the RS and control groups	Micro-CT showed that the bone tunnel area of RS and ARS groups was significantly smaller than those of the control group; Real-time polymerase chain reaction showed that BMP-2 and osteopontin expression levels at the interface in the RS and ARS groups were higher than those of the control group, and collagen I expression level of the ARS group was significantly higher than those of the RS and control groups.
	Hu et al., 2018	CS (collage-silk) (*n* = 10); CSL (collage-silk with LSPCs injection) (*n* = 15); CSF (collage-silk-SDF-1) (*n* = 10); CSFL (collage-silk-SDF-1 with LSPCs injection) (*n* = 15);		N/A	The CSFL group exhibited enhanced maturation of ACL tissue and improved bone tunnel healing	N/A	N/A
	Li et al., 2017	Control group (*n* = 48); Simplex fibrous membrane of PLLA (SFM) group (*n* = 48); dual-layer organic/inorganic flexible bipolar fibrous membrane (BFM) group (*n* = 48)	4, 8, 12 weeks	None	BFM significantly increased the area of glycosaminoglycan staining at the tendon–bone interface and improved collagen organization when compared to the simplex fibrous membrane (SFM) of PLLA.	The BFM group had a greater ultimate load-to-failure and stiffness than the SFM group at 12 weeks after surgery	Implanting the bipolar membrane also induced bone formation and fibrillogenesis as assessed by micro-CT.
	Chung et al., 2017	Reconstructed animals (*n* = 3); Non-operated control animals (*n* = 3)	6 weeks	N/A	In reconstructed animals, tissue infiltration throughout the entire scaffold and tissue ingrowth and interlocking within the bone tunnels.	N/A	N/A
	Lee et al., 2017	Saline only (control) (*n* = 12); Collagen gel without rhBMP-2(*n* = 12); rhBMP-2-conjugated collagen gel(*n* = 12)	3, 6 weeks	N/A	Fibrocartilage and new bone are formed at the interface at 6 weeks after injection of rhBMP-2	N/A	The micro-CT scan showed that spotty calcification appeared and enthesis-like tissue was produced successfully in the tendon at 6 weeks after injection of rhBMP-2
	Chou et al., 2016	Control (*n* = 24); PLGA/collagen nanofibrous membrane (*n* = 24)	16 weeks	N/A	The adequate biocompatibility of the PLA bolt on a medial cortex with progressive bone ingrowth and without tissue overreaction	The average maximal failure loads in PLGA/collagen nanofibrous membrane group was significantly higher than that in cintrol group	N/A
	Han et al., 2015	PCL/nHAp/Col group (*n* = 6); PCL group (*n* = 6)	4, 8 weeks	N/A	The scar tissue thickness was clearly smaller in the PCL/nHAp/Col group compared with the control group, and new bone tissue could be seen at the interface in PCL/nHAp/Col group	The failure load and the average stiffness was significantly higher in PCL/nHAp/Col group than in PCL control group	N/A
	Bi et al., 2015	Scaffold group (*n* = 20); Autograft group (*n* = 20)	4, 16 weeks	None	Abundant fibroblast-like cells were found in the core of the scaffold graft, and tenascin-C was strongly positive in newly regenerated tissu in the scaffold group, similar to the autograft group.	The failure load in the scaffold group was significantly higher than that in the autograft group at 4 weeks postoperatively. At week 16, the stiffness in scaffold group was significantly greater than that of the autograft group	Micro-CT scan found that obvious signals suggesting newly formed mineralized tissue were detected in the bone tunnels of both groups, and the average bone tunnel area in the scaffold group was significantly smaller than that in the autograft group
	Kovacevic et al., 2015	Control (*n* = 19); Scaffold only (*n* = 19); Three PDGF doses (30, 100, 300 μg/mol/L) on the collagen scaffold (*n* = 19)	5, 28 days	None	rhPDGF-BB delivery on a scaffold demonstrated a dose-dependent response in cellular proliferation and angiogenesis compared with the control and scaffold groups at 5 days, and had no effect on increasing fibrocartilage formation or improving collagen fiber maturity compared with controls at 28 days.	The control group had higher tensile loads to failure and stiffness than all the groups receiving the scaffold	N/A
	Zhao et al., 2015a	Transosseous repair (*n* = 48); PCL scaffolds (*n* = 48) PCL-CS scaffolds (*n* = 48)	2, 4, 8 weeks	N/A	The PCL-CS scaffolds enhanced new bone formation (mineralization) and collagen and glycosaminoglycan expression (major components of extracellular matrix) compared to the PCL scaffolds	The torn tissues at the tendon–bone insertion site regenerated with the PCL-CS scaffolds showed higher strength, failure strain and stiffness compared to those repaired using only the PCL scaffolds	N/A
	Zhao et al., 2015b	Transosseous repair (*n* = 48); PLLA membranes (*n* = 48); Gelatin-PLLA membranes (*n* = 48)	2, 4, 8 weeks	None	Gelatin-PLLA membranes have excellent biocompatibility and biodegradability, and significantly increased the area of glycosaminoglycan staining and improved collagen organization compared with the control group	Gelatin-PLLA group had a greater ultimate load to failure and stiffness than the control group	N/A
	Li et al., 2014	N/A	3 months	One animal with minor damage upon TCP insertion	There was a robust histological transition between regenerated fibrous tissue and the margins of the bone tunnel in Silk–TCP–PEEK, which had histological features similar to the native ACL to bone insertion	N/A	N/A
	Jiang et al., 2014	PET group (*n* = 24); PET + SF group (SF-coating group) (*n* = 24); PET + SF + HAP group (combined HAP- and SF-coating group) (*n* = 24)	4, 8 weeks	N/A	New bone tissue formation was only found in the PET + SF + HAP group, the PET fibers were almost completely encircled by collagen, and the width of the graft–bone interface was narrower than that in the other two groups	The mean load to failure and the stiffness value of the PET + SF + HAP group was higher than those of the PET group and the PET + SF group	The mRNA level of BMP-7 in the PET + SF + HAP groups was significantly higher than those in the other two groups
	Zhao et al., 2014	Transosseous repair (*n* = 48); PLGA membranes (*n* = 48); bFGF–PLGA membranes (*n* = 48)	2, 4, 8 weeks	None	Electrospun fibrous membranes have excellent biocompatibility and biodegradability, and significantly increased the area of glycosaminoglycan staining at the tendon–bone interface compared with the control group, and bFGF–PLGA significantly improved collagen organization	The electrospun fibrous membrane groups had a greater ultimate load-to-failure and stiffness than the control group, and the bFGF–PLGA membranes had the highest ultimate load-to-failure, stiffness, and stress of the healing enthesis	N/A
	Loeffler et al., 2013	Group I (control without surgery); Group II (surgical defect in the rotator cuff only); Group III (surgical defect with suture repair only); Group IV (surgical defect and repair with sponge only); Group V (surgical defect and repair with sponge loaded with cells)	3, 6, 12 weeks	None	The cellularity, inflammation, vascularity, and collagen organization increased in all repaired groups, and collagen organization at 12 weeks in Group V increased to improve healing with cells	N/A	N/A
	Vavken et al., 2012	ACL reconstruction (*n* = 8); Enhanced ACL repair (*n* = 8); ACL transection (*n* = 8);	15 weeks	None	N/A	There were no significant differences between bioenhanced ACL repair and ACL reconstruction.	N/A
	Li et al., 2012	Control group; LBL group	4, 8 weeks	N/A	New bone formed at the graft–bone interface in the LBL group, and the newly formed bone was similar with the host bone, and the interface between graft and host bone became narrow	The mean load-to-failure and mean stiffness for the LBL group was higher than that of the control group at 8 weeks	N/A
	Zhang et al., 2011	Control group (*n* = 17); Fibrin glue-BMP group (*n* = 17); RBX (recombined bone xenograft) group (*n* = 17)	2, 6, 12 weeks	N/A	The interface of fibrin glue-BMP developed new cartilage, and the interface of RBX had large areas of chondrocyte-like cells, bone formation and an immature neo-enthesis structure	The ultimate load of RBX group was higher than tfibrin glue-BMP group and control group	Micro-CT examination showed the value of bone mineral density in RBX group was significantly higher compared to fibrin glue-BMP group and control group at 12 weeks
	Fleming et al., 2009	Control group (*n* = 7); Experimental Group (*n* = 7)	15 weeks	None	Although cellular and vessel infiltration were observed in the grafts of both groups, regions of necrosis were present only in the standard ACL reconstructed group.	The normalized yield and maximum failure loads of the CPC group were higher than the standard ACL reconstructed group	N/A

*PGA, polyglycolic acid; PLGA, poly lactide-co-glycolide; BMP, bone morphogenetic protein; MSC, mesenchymal stem cell; PCL, polycaprolactone; KGN, kartogenin; SDF, stromal cell-derived factor; LSPCs, ligament-derived stem/progenitor cells; PDGF, platelet-derived growth factor; CS, chitosan; PLLA, poly L-lactide; PET, polyethylene terephthalate; TCP, tricalcium phosphate; PEEK, polyether ether ketone; ELAC, electrochemically aligned collagen; ACL, anterior cruciate ligament; PLA, polylactic acid; HAp/HA, hydroxyapatite; SF, silk fibroin; A-TSA, well-aligned Trichostatin A-laden fiber mats; A, aligned PLLA fibers; R-TSA, random fibers with TSA-loading; R, random fibers without TSA-loading*.

## Discussion

With the development of biomaterials science, the tissue engineering scaffold plays an extremely important role in tendon-bone healing. This systematic review has proved the efficacy and safety of synthetic scaffolds *in vivo*, which can be enhanced by biological interventions. However, the previous researches are mainly focused on animal models, not human trials which limits the clinical application of these scaffolds. In this review, we provide a descriptive summary of 27 articles related to the use of tissue engineering scaffold especially biomaterials in tendon-bone healing to better guide the clinical practice.

### Tendon-Bone Healing Process

Normal tendon-bone junctions are divided into indirect insertion and direct insertion (Mochizuki et al., 2006, 2014; Iwahashi et al., 2010; Sasaki et al., 2012). Indirect insertion refers to the thick fibrous tissue that directly connects the tendon or ligament to the periosteum, such as the medial collateral ligament tibial point (Mochizuki et al., 2006, 2014; Sasaki et al., 2012). Direct insertion refers to “anchoring” soft tissue to the deep layer of bone through typical fibrous cartilage tissue (Iwahashi et al., 2010). Its direct embedding point is a four-layer tissue transformation area, including bone, calcified cartilage, non-calcified cartilage, and ligament tissues, such as anterior cruciate ligament and rotator cuff. At present, the related research on the surgical technique and fixation method of tendon or ligament reconstruction aims to achieve the maximum contact area between the tendon and bone during reconstruction, ensure the stability and tightness of the contact surface, and minimize the influence of external forces. When the tendon is in contact with the bone, through direct or indirect tendon-bone healing, a normal tendon-bone interface is formed, a stable connection is established, and the reconstructed ligament functions. Direct tendon-bone healing refers to a tendon-bone connection with four-layer structure, which exists between the tendon and the tunnel opening after healing (Iwahashi et al., 2010; Sasaki et al., 2012). Indirect tendon-bone healing refers to the connection between tendons and bones through Sharpey-like fibers (Mochizuki et al., 2006, 2014). This type of healing takes a long time, and the tensile strength between tendons and bones after healing is lower than that of direct healing.

An animal experiment showed that (Weiler et al., 2002), after the tendon is implanted in the bone tunnel, it needs to go through the necrosis period, the proliferation period, and the ligamentization period to achieve true healing. The period of necrosis is also called early healing, and generally refers to the first 4 weeks after the implant is implanted. During this process, the graft has necrosis, and its mechanical strength is significantly lower than that of the implant. The proliferative phase refers to the remodeling, revascularization, and strong cell activity of the graft from 4 to 12 weeks after surgery. The ligamentization period refers to the period from the proliferation period to the end of graft remodeling, which can be as long as half a year to several years. Tabuchi et al. (2012) observed the tendon implanted in the rabbit bone tunnel for 26 weeks; the results showed that Sharpey-like fibers containing type III collagen fibers were gradually replaced by type I collagen from 12 weeks after surgery until the fibers in the graft formed a connection with Sharpey-like fibers at week 26.

### Biomaterials of Tendon-Bone Healing and Bone Tissue Engineering Scaffolds

Biomaterials of tissue engineering scaffold is an important and difficult point in the research of tissue engineering. Without suitable scaffolds, the seed cells will be lost and die. Tissue engineering scaffold materials should have good biocompatibility, biodegradability, three-dimensional structure, plasticity, and equivalent mechanical strength. It also has good surface activity, which is conducive to the adhesion of seed cells and provides a good microenvironment for the growth and reproduction of cells on the surface and secretion matrix. For tendon and tissue engineering scaffold materials, the most researched at present are natural materials, synthetic materials, copolymers, and composite materials. The following are commonly used and investigated in this review.

#### Natural Materials

The excellent biocompatibility of natural polymers such as collagen and fibrin can significantly improve the interaction between materials and tissue cells (Doty et al., 2019). In our review, collagen is widely used and evaluated in tendon bone healing. It is the main component of extracellular matrix (ECM), which can be extracted from animal bones and fascia through digestion, hydrolysis, and other processes (Shi et al., 2019; Mohammed et al., 2020). In the process of evolution, collagen retains the original amino acid sequence, and the scaffold material made by it has non-antigenicity, good biocompatibility *in vivo*, and permeability (Dunshee et al., 2020). Because the tissue composition of tendons is mainly composed of collagen fibers, the large fiber bundles are arranged in parallel, and their direction is consistent with the traction force they bear. Collagen fiber has toughness and strong traction resistance; in addition, the cell adhesion signal sequence contained in it can also guide the specific identification of the scaffold material by the cells. Collagen fiber has toughness and strong traction resistance (Sharma et al., 2019; Tonndorf et al., 2020). After years of development, its preparation method has been quite mature. It has now been certified by the US Food and Drug Administration (FDA) and can be successfully used in ECM scaffolds for tissue-engineered tendons. Bellincampi et al. (1998) inoculated autogenous tendon cells into collagen scaffolds and implanted them in rabbit knee joints and subcutaneously. The complex was still visible after 8 weeks. Young et al. (1998) used autologous bone MSC to adsorb on collagen gel and planted the compound on the joint of tendon. It was found that the tendon treated with MSC was thicker than the control group, and the collagen fiber assembly, seam characteristics, and load performance were better than the control group. In the included studies, the collage has been used as tissue engineering scaffold for tendon bone healing in many animal models (Fleming et al., 2009; Vavken et al., 2012; Bi et al., 2015; Kovacevic et al., 2015; Lee et al., 2017; Hu et al., 2018; Learn et al., 2019; Zhu M. et al., 2019), and its efficacy can be improved by utilizing a biofabrication process known as electrocompaction. Learn et al. (2019) had used electrochemically aligned collagen (ELAC) threads woven into biotextile scaffolds as grafts to repair critical infraspinatus tendon defects in New Zealand white rabbits, and it was found that woven ELAC scaffolds was intact and mechanically competent following 3 months of implantation and biological characteristics of tendon were present in the tissue around and within the scaffolds.

Acellular scaffolds play a unique role in promoting tendon bone healing. Lu et al., 2019 found an appropriate decellularization protocols for fibrocartilage tissue and prepared a novel book-shaped acellular fibrocartilage scaffold with cell-loading capability and chondrogenic inducibility for tissue-engineered fibrocartilage and bone-tendon healing. This screened scaffold alone could induce endogenous cells to satisfactorily regenerate fibrocartilage at 16 weeks, as characterized by fibrocartilaginous ECM deposition and good interface integration and may have further broad clinical applications in promoting bone-tendon healing. Then they creatively prepared two kinds of acellular scaffolds from bone or fibrocartilage tissue, namely book-type acellular bone scaffold (BABS) and book-type acellular fibrocartilage scaffold (BAFS). Histologically, both scaffolds well preserved the natural ECM structure without cellular components. *In vitro* studies have shown that BABS has a good ability of osteogenic induction, while BAFS has a good ability of cartilage induction. Using a rabbit partial patellectomy model, both BAFS and BABS can promote tendon-bone healing, while the BAFS was more conductive in tendon-bone healing than the BABS. This study may provide a valuable reference for screening the optimal tissue-engineering scaffold applied in tendon-bone healing (Lu et al., 2019).

#### Synthetic Materials

Synthetic materials like PLA, polyglycolic acid (PGA) possess three main structural forms (fiber scaffold, porous foam, tubular structure). The degradation products of PLA and PGA are lactic acid and glycolic acid, respectively, which are intermediate metabolic products of the tricarboxylic acid cycle. They have good biodegradability and compatibility and will not cause inflammation, immune reactions, and cytotoxic reactions. The most widely used biodegradable biomaterials have been widely used in tissue engineering such as bone, cartilage, blood vessels, nerves, and skin (Learn et al., 2019). Cao et al. (2002) inoculated tendon cells obtained from the tendon tissues of calf shoulders and knees on a cable-like PGA mesh scaffold and implanted them subcutaneously in nude mice after 1 week *in vitro* culture. It was found that at 12 weeks, tendon tissue similar to the normal tendon structure was formed, and it had a certain degree of biomechanical properties. Cao et al. (2002) also used the method of autologous tendon cells + PGA + biofilm wrapping to repair the 4 cm tendon defect in Leghorn muscles. The results showed that the implanted tissue-engineered tendon was small, similar to the normal tendon only in general morphology and histology, and its biomechanical properties also reached 83% of the normal tendon.

Moreover, PCL, as a synthetic material, can also be used as scaffold to repair the defect of tendon bone, and many studies also have proved the application of PCL (Han et al., 2015; Zhao et al., 2015b; Han F. et al., 2019; Li et al., 2019). PCL is a kind of thermoplastic crystalline polyester obtained by ring-opening polymerization of caprolactone with diol as initiator, containing many methyl groups, so it has hydrophobicity. In addition, its degradation rate is lower than other aliphatic polyesters (Hagg et al., 1991; Crair et al., 1998). Han F. et al. (2019) have found that PCL scaffold can be loaded with biological interventions such as BMP-2 and SDF-1α to form fibrous scar tissue and new bone for tendon bone healing. On other hand, the use of PET is also investigated in previous studies (Li et al., 2012; Jiang et al., 2014). PET is a crystalline-saturated polyester with excellent physical and mechanical properties in a wide temperature range. Earlier, PET was mainly used in the preparation of LARS artificial ligament. Because of its poor hydrophilicity and lack of bone conductivity, it is not conducive to the growth of autogenous bone tissue, thus affecting the tendon-bone healing of the tendon in the bone tunnel and inducing ligament loosening which causes operation failure in the middle and long term after operation (Thian et al., 2006). At the same time, fibroblasts and synoviocytes cannot grow on the surface of ligament joint cavity and wrap LARS ligament to induce autologous tissue growth as the poor cytocompatibility of PET material, which will eventually lead to wear and fatigue fracture, and the shedding of wear particles will cause serious synovitis of knee joint. To solve this problem, Jiang et al. (2014) combined silk fibroin (SF) and hydroxyapatite (HAp/HA) coating by biomimetic route on the surface of PET artificial ligament; the results showed that this combination could induce graft osseointegration in the bone tunnel. Moreover, Li et al. (2012) had proven that surface coating with an organic layer-by-layer self-assembled template of chitosan and hyaluronic acid on a PET artificial ligament could be designed to promote and enhance the graft-to-bone healing after artificial ligament implantation in a bone tunnel.

#### Copolymer

The PLA and PGA copolymers include PLGA, poly(d-lactic acid) (PDLA), poly (l-lactic acid) (PLLA), poly(d,l-lactic acid) (PDLLA) (Kikuchi et al., 2018). Since PLGA has excellent biocompatibility and tunable mechanical and degradation properties, it is the most frequently used applications of PLA and PGA in tendon tissue engineering. PLGA not only has good biocompatibility but can also induce the upregulation of certain genes (Fujimaki et al., 2020). The degradation rate can also be controlled by changing the ratio of PLA to PGA and combining the high degradation rate of PGA and the high strength of PLA. Therefore, PLGA can also be used as a cell scaffold for artificial tendons. Fujimaki et al. (2020) and Lim et al. (2019) used PLA and PGA compound PLGA as a stent and implanted a 10-mm defect into the autogenous tendon. The control group was simply replanted with PLGA. Examination after 4 weeks revealed that the cell content was significantly reduced compared with that at 2 weeks, and collagen fibers I and III were formed, and the experimental group was more obvious than the control group. The material was basically degraded at 8 weeks, and the defect was repaired well at 12 weeks, with no lymphocyte infiltration. The biomechanical strength of the experimental group was significantly higher than that of the control group, close to normal tendons. Cai et al. (2018) prepared a dual-layer aligned-random nanofibrous scaffolds SF/P(LLA-CL) using SF-blended poly(l-lactic acid-co-e-caprolactone) (PLLA-CL), which could effectively augment the tendon-to-bone integration and improve gradient microstructure in a rabbit extra-articular model by inducing the new bone formation, increasing the area of fibrocartilage, and improving collagen organization and maturation.

#### Composite Materials

Natural materials such as collagen have good biocompatibility, but there are defects such as poor mechanical properties, very fast degradation, and poor processing performance. Synthetic materials such as high-molecular materials have defects such as low degradation rate, inflammation caused by acidic degradation products, and small mechanical properties. Thus, it is difficult for a single type of material to meet the requirements of extracellular matrix materials for tendon-bone healing. These problems can be solved by the principles and methods of composite materials. That is to say, two or more kinds of biomaterials with complementary characteristics are combined in a certain proportion and manner in order to construct a new composite material that can meet the requirements. Researchers combined several single materials through appropriate methods, comprehensively considered the advantages and disadvantages of organic materials and inorganic materials, and complemented each other to form organic/inorganic composite materials, which achieved good results in practical applications. Devin et al. (1996) composed HAp and PLA/PGA copolymer (50:50) into a porous composite matrix material and found that the compressive elastic modulus of the composite increases with the HAp composition. Therefore, the introduction of calcium-phosphorus ceramics into the PLA/PGA copolymer can improve the shortcomings of poor mechanical properties, fast degradation rate, and weak bone binding ability of the PLA/PGA copolymer. Besides, research by Serre et al. (1998) confirmed that porous materials composed of type I collagen, chondroitin sulfate, and HAp/HA can promote the attachment and growth of osteoblasts and promote the calcification of their secreted matrix. Li et al. (2017) developed a dual-layer organic/inorganic flexible bipolar fibrous membrane (nHA-PLLA) to repair the defect of upraspinatus tendon in New Zealand rabbit, which could act as a bridge between the repaired tendon and footprint, affecting the healing process in multiple ways. In other studies, Chung et al. (2017) successfully fabricated a biodegradable and synthetic tri-component graft consisting of porous poly(1,8-octanediol-co-citric acid)-hydroxyapatite nanocomposites (POC-HA) and poly(l-lactide) (PLL) braids, Han et al. (2015) developed a biomimetic nanofiber membrane of polycaprolactone/nanohydroxyapatite/collagen (PCL/nHAp/Col), and Li et al. (2014) prepared a silk-TCP-PEEK scaffold of silk, TCP, and polyether ether ketone (PEEK) to improve the healing and regeneration process of the tendon-bone defect. Zhang et al. (2018) found that trichostatin A (TSA) incorporated aligned fibers of PLLA had an additive effect in directing tenogenic differentiation and confirmed that composite scaffold promoted the structural and mechanical properties of the regenerated Achilles tendon, which provides fresh insights into the regulation of tendon differentiation and a clinically applicable therapeutic approach for tendon regeneration.

#### Bionic Scaffold

Bionic scaffolds have been of knee interest in tissue engineering which are sufficient for tissue regeneration. Liu et al. (2017) developed a bionic random-aligned-random-tendon ECM composite scaffold for reconstruction of the soft tissue-bone junction in rabbit model. Microcomputed tomography (micro-CT) and histological analysis showed that the bionic scaffold enhanced tendon bone healing and fibrocartilage formation. These results demonstrated that the bionic scaffold could be a promising scaffold for ligament/tendon-bone junction repair. Lipner et al. (2015) used bionic scaffolds implanted with pluripotent cells to promote tendon-to-bone healing by promoting collagen deposition and bone formation. Adipose-derived stromal cells were implanted into the repair site of rat rotator cuff model to construct nanofiber polylactic acid-glycolic acid copolymer scaffolds with different mineral content gradients. Histologically, the healing interface of all groups was mainly fibrovascular scar reaction. The results showed that the tendon-to-bone healing was dominated by scar formation, which prevented any positive effect of the implanted biomimetic scaffold. In a word, the role of biomimetic scaffold in promoting tendon bone healing is not very clear, and more studies are needed to prove its positive effect.

### Preparation Method of Scaffolds

Porous and three-dimensional (3D) scaffolds have been extensively used as biomaterials in the field of tissue engineering *in vitro* study of cell-scaffold interactions. There are many methods for preparing porous materials, such as particle filtration method, melt molding method, emulsion freeze-drying method, high-pressure gas expansion method, fiber three-dimensional interweaving method, phase separation method, etc. (Freedman and Mooney, 2019). Some scholars try to use two or more methods together, or improve existing methods, optimize process parameters, in order to obtain a better pore structure. Harris et al. (1998) used the gas-foaming method combined with the particle filtration method to prepare the stent material with open large pores, which effectively overcome the disadvantages of closed pores. Mikos et al. (1993) applied lamination technology to prepare PLA and PGA into a three-dimensional polymer foam with a certain shape. The micropores of the laminated layers communicate with each other to form a continuous cell structure. These are conducive to cell growth, survival, and proliferation. An intelligent processing technology called rapid prototyping has achieved rapid development in this field (Szlazak et al., 2019). The technology is based on the principles of dispersion and stacking. The three-dimensional model is constructed by performing layer processing on the images obtained by CT or magnetic resonance scanning of the human body, and then layered and sliced. Finally, the STL format file is transferred to the rapid prototyping machine for processing, and the various cross-sectional contours are formed by means of hot melting and cutting, and gradually stacked into a three-dimensional part. This method can be tailored according to the requirements of different patients and has the characteristics of being fast and flexible, so it has great advantages in the processing of tissue engineering scaffolds (Duty et al., 2007; Ravichandran et al., 2017).

### Biodegradation

In the past 10 years, the rapid degradation of various materials in the human body is still a major problem hindering its clinical application. Because the interface of tendon bone connection involves two different biological structures, the ideal biomaterial used for tendon bone healing should have a higher standard. In other words, it is necessary to ensure that the stress at the bone-tissue interface can be reduced while matching with the degradation rate during bone healing period, and the enzymatic hydrolysis *in vivo* at the end of the tendon can be resisted while the degradation products will not cause changes of the internal environment pH. Therefore, the widely developed biphasic and triphasic stents are very competitive (Atesok et al., 2016; Tang et al., 2020; Zhou et al., 2021). The degradation rate could be adjusted by ultrasonic pretreatment, and the electrostatic attraction or physical cross-linking between the molecules could be used to improve the stability of the materials and add cross-linking agents to make the internal molecular chains of the composites produce stronger cross-linking.

## Conclusion

Functional fibrocartilage regeneration is a choke point in tendon bone healing, and the currently available tissue-engineering strategies for fibrocartilage regeneration are insufficient because of a lack of appropriate scaffold that can load large seeding cells and induce chondrogenesis of stem cells. Numerous strategies have been employed to improve tendon bone junction healing, including delivery of stem cells, bioactive factors, and synthetic materials, but these are often inadequate at recapitulating the complex structure-function relationships at native tissue interfaces Based on the results of several studies, it is found that the challenges that may be faced in the clinical process of tendon bone healing include the following: the regeneration strategy may be overwhelmed by natural scar-mediated responses, BMP2 is not an effective growth factor to promote tendon bone healing, and scaffold materials may have a negative effect on tendon bone healing.

At present, except for the periosteum which has been used clinically and the curative effect is clear, the remaining methods to promote tendon-bone healing have no definite conclusions, and further research is needed to provide a more stable healing effect after ligament or tendon repair and reconstruction. Although a number of tissue engineering scaffolds have been developed and investigated, whose efficacy and safety has been proved and can be enhanced by biological interventions in this review, the researches are mainly focused on animal models which are with limitations in clinical application. On the other hand, tendon tissue engineering has high requirements for scaffold materials, and the development and research of composite materials will continue to be a hotspot for future research. Therefore, substantial clinical trials remain to be done, and continued progress in overcoming current tissue engineering challenges should allow for successful clinical practice, which is also one of the main directions of tissue engineering material research and development in the future.

## Data Availability Statement

The original contributions presented in the study are included in the article/supplementary material, further inquiries can be directed to the corresponding authors.

## Author Contributions

ZM, BF, and XW: substantial contributions to the conception or design of the work, or the acquisition, analysis, or interpretation of data for the work. XH, JG, ZS, BX, MY, and ZC: drafting the work or revising it critically for important intellectual content. DJ and JY: final approval of the version to be published, agreed to be accountable for all aspects of the work in ensuring that questions related to the accuracy or integrity of any part of the work are appropriately investigated and resolved.

## Conflict of Interest

The authors declare that the research was conducted in the absence of any commercial or financial relationships that could be construed as a potential conflict of interest.
